# A role for thioredoxin-interacting protein (Txnip) in cellular creatine homeostasis

**DOI:** 10.1152/ajpendo.00637.2012

**Published:** 2013-05-28

**Authors:** Sevasti Zervou, Tanmoy Ray, Natasha Sahgal, Liam Sebag-Montefiore, Rebecca Cross, Debra J. Medway, Philip J. Ostrowski, Stefan Neubauer, Craig A. Lygate

**Affiliations:** ^1^Division of Cardiovascular Medicine, Radcliffe Department of Medicine, University of Oxford, Headington, Oxford, United Kingdom; and; ^2^Bioinformatics and Statistical Genetics Core, Wellcome Trust Centre for Human Genetics, Headington, Oxford, United Kingdom

**Keywords:** cardiomyocytes, redox

## Abstract

Creatine is important for energy metabolism, yet excitable cells such as cardiomyocytes do not synthesize creatine and rely on uptake via a specific membrane creatine transporter (CrT; SLC6A8). This process is tightly controlled with downregulation of CrT upon continued exposure to high creatine via mechanisms that are poorly understood. Our aim was to identify candidate endogenous CrT inhibitors. In 3T3 cells overexpressing the CrT, creatine uptake plateaued at 3 h in response to 5 mM creatine but peaked 33% higher (*P* < 0.01) in the presence of cycloheximide, suggesting CrT regulation depends on new protein synthesis. Global gene expression analysis identified thioredoxin-interacting protein (Txnip) as the only significantly upregulated gene (by 46%) under these conditions (*P* = 0.036), subsequently verified independently at mRNA and protein levels. There was no change in Txnip expression with exposure to 5 mM taurine, confirming a specific response to creatine rather than osmotic stress. Small-interfering RNA against Txnip prevented Txnip upregulation in response to high creatine, maintained normal levels of creatine uptake, and prevented downregulation of CrT mRNA. These findings were relevant to the in vivo heart since creatine-deficient mice showed 39.71% lower levels of Txnip mRNA, whereas mice overexpressing the CrT had 57.6% higher Txnip mRNA levels and 28.7% higher protein expression compared with wild types (mean myocardial creatine concentration 124 and 74 nmol/mg protein, respectively). In conclusion, we have identified Txnip as a novel negative regulator of creatine levels in vitro and in vivo, responsible for mediating substrate feedback inhibition and a potential target for modulating creatine homeostasis.

creatine (Cr) entry into cells occurs via the 12 transmembrane domain creatine transporter (CrT; SLC6A8), a family member of the Na^+^Cl^−^-dependent neurotransmitter transporters (16, 34, 37). Most cells, including cardiomyocytes, do not synthesize Cr de novo and rely on entry of Cr through CrT. The importance of tight regulation of myocardial Cr has previously been described in detail (22, 37, 41). Recently, moderate increases in intracellular Cr and phosphocreatine (PCr) concentrations have been shown to protect against ischemia-reperfusion injury in the heart (21), whereas large increases (>2-fold) are associated with hypertrophy and impaired contractility (40).

Multiple studies have investigated mechanisms for controlling Cr entry into cells via the CrT, but a clear consensus has yet to emerge. For example, AMP-activated protein kinase reduces CrT expression in kidney epithelial cells (17) but increases Cr uptake in cardiomyocytes overexpressing CrT (7). Studies in *Xenopus* oocytes expressing CrT cDNA have shown that serum- and glucocorticoid-inducible kinases (SGK1 and SGK3) increase CrT activity, acting downstream from the mammalian target of rapamycin (35, 39). However, the physiological significance of these artificial systems is questionable, and phospho-SGK1 was undetectable in hearts from Cr-deficient mice despite a large increase in Cr uptake capacity (41). Growth hormone increased CrT gene expression in vivo in rats postmyocardial infarction; however, this did not result in elevated myocardial Cr levels (27). Perhaps most convincingly, insulin and insulin-like growth factor-I positively modulate CrT activity, enhancing Cr uptake in skeletal muscle (11, 15) and in G8 myoblasts in vitro (26).

CrT is also subject to posttranslational modifications such as phosphorylation at Tyr^416^, resulting in altered CrT activity during starvation and sepsis (37, 22, 44, 51). In addition, CrT is a glycoprotein with two consensus *N*-glycosylation sites on the second extracellular loop. Mutations at either site decrease CrT activity and are potentially implicated in CrT surface trafficking (38).

So far, the one clear regulator of CrT activity is feedback inhibition by Cr itself (41, 5, 8, 10). Feeding rats excess dietary Cr reduces Cr uptake capacity in the heart while giving β-guanidinopropionic acid to deplete intracellular Cr results in increased uptake capacity (5), an effect also observed in human muscle cells and G8 myoblasts (19). Similarly, Cr-deficient mice display a sevenfold increase in Cr uptake kinetics, which is abolished by Cr feeding (41). However, the molecular mechanisms for downregulation of CrT by Cr have yet to be elucidated.

One important clue is that exposure of rat and human muscle cells to saturating (mM) levels of Cr results in decreased CrT activity, but that this can be blocked by incubation with cycloheximide (CHX), a widely used protein synthesis inhibitor (19). This infers synthesis of new protein(s) in response to high Cr, which effectively act to inhibit CrT activity (19).

In this study, we therefore sought to identify the endogenous inhibitor of CrT activity. To this end, we used an in vitro system of CrT overexpression and followed a global gene array approach to identify candidate CrT inhibitors. Thioredoxin-interacting protein (Txnip), known as vitamin D_3_-upregulated protein, with roles in redox and metabolic regulation was the only molecule that increased at the gene and protein levels in cells treated with saturating levels of Cr. The role of Txnip in regulation of CrT activity was verified in vitro, followed by an investigation on the induction of Txnip by altered Cr in vivo, using mouse models of either depleted or enhanced myocardial Cr levels.

## MATERIALS AND METHODS

### 

#### Chemicals.

All chemicals were supplied by either Sigma-Aldrich (Poole, UK), Tocris Bioscience (Bristol, UK), or VWR (Lutterworth, UK). Tissue culture media were purchased from Lonza (Slough, UK), PAA (Yeovil, UK) or Sigma-Aldrich. Compounds were purchased from the following companies, respectively, creatine monohydrate and CHX (Sigma); DMSO used at 0.1–0.5% (Fisher Scientific). Small-interfering RNA (siRNA) oligonucleotides were purchased from Thermo Fisher Dharmacon (Loughborough, UK).

#### In vitro cell culture system and Cr uptake assays.

3T3 Tet “Off” mouse fibroblasts were transfected to stably express a full-length cDNA clone of the rabbit *CrT* gene (accession no. X67252) with a COOH-terminal hemagglutinin epitope tag (3T3-CrT cells; see Ref. 21). Genetic and functional upregulation of CrT was previously verified in these 3T3-CrT cells. A dramatic increase of CrT mRNA was observed compared with untransfected Tet Off cells (∼80-fold). Cr uptake follows Michaelis-Menten kinetics; 3T3-CrT showed 11-fold upregulation of CrT activity compared with 3T3 Tet Off cells (data not shown).

The method of Cr uptake was adapted from Walzel et al. (43). 3T3-CrT cells were grown in 24-well tissue culture plates (Greiner Bio One, Stonehouse, UK) to 70–80% confluence in complete DMEM. Media were aspirated from the wells and replaced by Cr-containing media (at 250 μM) and then “spiked” with 0.0074 MBq of [^14^C]Cr. The culture plates were incubated for 1 h at 37°C with 95% O_2_ and 5% CO_2_. Following incubation, the radioactive media were removed, and the wells were washed three times in 1 ml phosphate-buffered saline (PBS). Cells were then permeabilized in 0.5% Triton X-100 and combined with 10 volumes of scintillation cocktail (Fluoransafe XE; VWR) in the dark at room temperature for 1 h. Samples were then analyzed in a scintillation counter (Beckman Coulter UK, High Wycombe, UK) against standards containing known quantities of spiked media. The values were compared with those of the spiked media, and the uptake levels were determined, expressed as nanomoles Cr per well per hour. For the CHX experiment, cells were exposed to two doses of Cr, saturating (5 mM) and low (250 μM), in the presence or absence of CHX (10 μg/ml) for 1–6 h to determine the optimal incubation time. In this case, spiking with [^14^C]Cr followed the 1- to 5-h incubation with Cr with or without CHX. The same is valid for all experiments when cells were treated with 5 mM Cr for 3 h before a Cr uptake assay.

#### High-pressure liquid chromatography.

Mouse left ventricular (LV) samples were excised and snap-frozen in liquid nitrogen before homogenization and analysis. 3T3-CrT cells in six-well plates were treated with 5 mM creatine monohydrate in growth media for 3 h. Following this, cells were detached from the culture plates as for passaging, using standard Trypsin/Versene solution, and pellets from each well were generated after centrifugation at 1,000 rpm for 5 min at room temperature. Cell pellets (including nontreated) were washed two times with PBS at room temperature, including two subsequent centrifugations as above. All remaining supernatant was removed after the final wash, and cell pellets were snap-frozen in liquid nitrogen and stored at −80°C before analysis by high-pressure liquid chromatography (HPLC). The method was adapted from Neubauer et al. (24). Each frozen and powdered LV sample or cell pellet was homogenized in a previously chilled stainless steel percussion mortar, in 0.4 N perchloric acid on ice, and an aliquot of the homogenate was removed for protein determination. The homogenate was neutralized and centrifuged at 5,500 rpm for 5 min. The filtered supernatant was used for measuring total Cr (i.e., PCr + Cr) by HPLC, using a Supelcosil LC-18-T, 5 μM (Supelco) chromatography column. Mobile phase was 3.5% acetonitrile, with 215 mM KH_2_PO_4_ and 2.3 mM tetrabutylammonium hydrogen sulfate (C_16_H_37_NO_4_S). Separation was performed at 30°C, at a flow of 0.7 ml/min. For tissue homogenates, Cr values were normalized over protein, following noncollagen protein determination by the method of Lowry et al. (20).

#### Transgenic mouse models.

LV tissue was obtained from two mouse strains after death by cervical dislocation. For this purpose, male mice at 8 wk of age were used. Mice with deletion of the second essential enzyme for Cr biosynthesis (guanidinoacetate methyltransferase knockout, GAMT^−/−^) (31) have an absolute whole body Cr deficiency when fed a standard Cr-free diet. Mice with myocardial CrT overexpression (CrT-OE) have elevated levels of cellular Cr in the heart, as previously reported (42, 29). Both strains have been backcrossed with C57BL/6J for >10 generations, and age-matched wild-type littermates were used as controls.

#### mRNA isolation and global gene array.

For gene arrays, 3T3-CrT cells were treated with 5 mM creatine monohydrate for 3 h before total RNA isolation (Qiagen RNEasy kit; Qiagen, Crawley, UK) according to the manufacturer's instructions and as described before (47). For qRT-PCR, the quality and integrity of the RNA samples were evaluated by using the Nanodrop (Agilent, Wokingham, UK) while, for Gene Arrays, RNA Integrity Numbers (RIN) were estimated by a Bioanalyzer (Agilent). RNA samples were confirmed to have a RIN ∼10 and used for subsequent use in Gene Array analysis. A genomewide analysis of gene expression was carried out in six RNA samples from cultured cells (*n* = 3 for “no supplement” and *n* = 3 for 5 mM Cr) using Illumina's Mouse WG6v2 Expression BeadChip. Total RNA was converted into labeled cRNA and used for hybridization. The hybridized and washed chips were then scanned using an Illumina Bead Array Scanner. The raw data were exported from the Illumina GenomeStudio software (version 1.0.6) for further processing and analysis. Raw signal intensities were background corrected using array-specific measures of background intensity based on negative control probes, in the R statistical software (version 2.13) (30) with BioConductor packages (9), before being transformed and normalized using the “vsn” package (12). Quality control analyses did not reveal any outlier samples. The dataset was then filtered to remove probes not detected (detection score <0.95) in any of the samples, resulting in a final dataset of 20,308 probes. Differential expression between the experimental groups was assessed by generating relevant contrasts corresponding to the two-group comparison and was performed using the Linear Models for Microarray Analysis package (36). Raw *P* values were corrected for multiple testing using the false discovery rate controlling procedure of Benjamini and Hochberg (4). Significant probe lists were then annotated using the relevant annotation file (MouseWG6_V2_0_R0_11278593_A) that was downloaded from the Illumina website (http://www.illumina.com) for further biological investigation. Gene array data were deposited on the NCBI Gene Expression Omnibus (www.ncbi.nlm.nih.gov/geo/; accession: GSE42356; ID:200042356).

#### qRT-PCR.

Total RNA was prepared from cells as described above, whereas mouse LV RNA was extracted using Trizol reagent (Invitrogen) and a phenol/chloroform step before purification by the Qiagen RNeasy Kit as above. One nanogram per reaction of total RNA was used to reverse transcribe to cDNA and amplify in one step using the Bio-Rad CFX96 machine using the iScript one-step reagent, according to the manufacturer's protocol (Bio-Rad, Hemel Hempstead, UK). The oligonucleotides used are listed on Table 1. For quantification purposes, mRNA levels were normalized over the reference gene 36B4 (1) and the 2^−ΔΔC_T_^ method (18).

**Table 1.** List of oligonucleotides used in qRT-PCR

Accession No.	Name	Forward	Reverse
NM_001009935	*Txnip*	5′-GGGGGCAGCCTACAGCAGGT-3′	5′-GGGGGGCTGGCTGGGGCG-3′
n/a	*CrT*	5′-ACTGTGTGGAGATCTTCCGC-3′	5′-CAGCAAGCTGGTCACATGTG-3′
NM_007475	*36B4*	5′-AGATTCGGGATATGCTGTTGG-3′	5′-TCGGGTCCTAGACCAGTGTTC-3′

Oligonucleotide sequence as used in quantitative RT-PCR. Accession numbers (No.) are shown for thioredoxin-interacting protein (Txnip) and 36B4, whereas “n/a” stands for the total endogenous creatine transporter (CrT, a common sequence in overexpressed rabbit and the endogenous mouse CrT; as described in Ref. 41).

#### Protein extraction and immunoblotting.

Cells were harvested using ice-cold RIPA buffer (Sigma) containing Complete Protease Inhibitor Cocktail (Roche), PMSF (Santa Cruz Biotech), and 1 mM DTT (Sigma). The crude extract was sonicated on ice and then centrifuged at 13,000 rpm for 10 min at 4°C before removing the supernatant and storing at −20°C. The samples were then normalized for protein content following the bicinchoninic acid assay (Pierce Thermo Scientific) and boiled in Laemmli buffer and reducing solution (Nupage; Invitrogen) for direct SDS-PAGE (2, 21). Twenty micrograms of protein were separated on precast 12% Tris glycine SDS-PAGE gels (Thermo-Pierce, Cramlington, UK) in 1× Tris-HEPES running buffer (Thermo-Pierce). Proteins were transferred to a polyvinylidene difluoride membrane (GE Healthcare, Amersham, UK) before blocking in 5% dry milk powder with 0.1% Tween 20 in PBS for 1 h and incubation in primary rabbit antibody against Txnip (Novex, #40-3700; Invitrogen) overnight at 4°C, followed by 1 h incubation at room temperature with appropriate peroxidase-conjugated secondary antibodies. Detection of immunoblot signals was performed using the ECL advance chemiluminescence kit (GE Healthcare) and a FluorChem 8800 imager (Alpha Innotech). For normalization purposes, the blots were stripped off the primary antibody and reprobed against α-actinin (Sigma) or β-tubulin (Abcam).

#### Experiments with siRNA.

3T3-CrT cells were grown to 60% confluency in 24-well plates before transfection in serum-free media, using a combination of 5 μM siTxnip oligo [single ON-TARGETplus siRNA against mouse Txnip (56338) open-reading frame; catalog no. J-040441-10-0005] and Dharmafect 1 transfection reagent (Thermo; Dharmacon) according to the manufacturer's manual. Forty-eight hours posttransfection, cells were exposed to 5 mM Cr for 3 h before a Cr uptake assay (described above). A replicate 24-well plate was used for subsequent RNA extraction, assessment of transfection efficiency by qRT-PCR, or for protein extraction and immunoblotting. Sham transfections were included as negative controls, using transfection reagent only (−siRNA). Positive control transfection of siRNA against cyclophilin B showed a drop in mRNA by 55.72% compared with the −siRNA sample and did not change either Txnip or CrT transcripts (data not shown).

#### Data analysis.

All samples were analyzed blind to genotype or treatment. Data are presented as means ± SE, and differences were considered significant when *P* ≤ 0.05. Cr uptake assays were analyzed by one-way ANOVA with Bonferroni's correction for multiple comparisons. Dunnett's posttest against control was applied when several concentrations or incubation times were being tested. Student's *t*-test was used to compare two groups of equal variances. The CHX experiment was analyzed by two-way ANOVA. The gene array experiment was analyzed as described above.

## RESULTS

### 

#### High Cr results in protein synthesis of a CrT inhibitor.

**Fig. 1.**
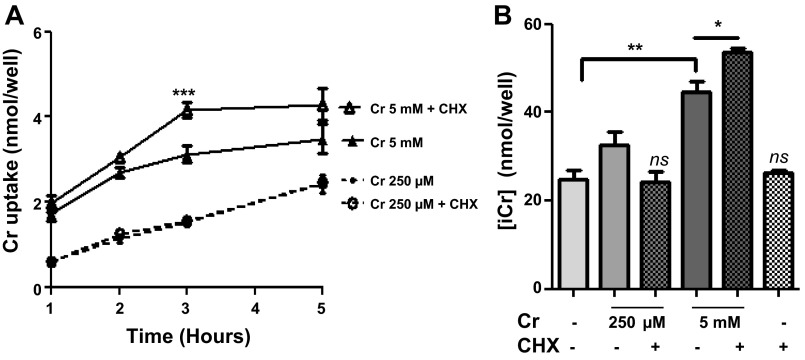
*A*: inhibition of protein synthesis by cycloheximide (CHX) increases cellular creatine (Cr) uptake. 3T3-CrT cells were exposed to CHX for 1–5 h in the presence of either 250 μM (low) or 5 mM (saturating) creatine concentration ([Cr]). Uptake at the low Cr concentration was unaffected by CHX, whereas CHX in the presence of saturating [Cr] increased uptake by 33% compared with 5 mM Cr alone (***P < 0.0001). The effect peaked at 3 h; *n* = 3/time point and per treatment. Summary of 4 independent experiments is shown here. Means ± SE per treatment were as follows: 250 μM, 1.420 ± 0.387; 250 μM + CHX, 1.456 ± 0.384; 5 mM, 2.754 ± 0.381; 5 mM + CHX, 3.364 ± 0.544. *B*: intracellular [Cr] increased from 24.69 ± 2.07 to 32.50 ± 2.97 with 250 μM Cr compared with a more pronounced increase (to 44.50 ± 2.47) with 5 mM Cr after 3 h. CHX decreased Cr uptake when coincubated with 250 μM but caused a further increase in intracellular creatine (i[Cr]) when supplemented with 5 mM Cr compared with Cr only. **P = 0.0081 and *P = 0.0257. ns, Nonsignificant.

Fibroblasts overexpressing the CrT were incubated in the presence of creatine monohydrate at 250 μM (low levels) and 5 mM (saturating levels) with Cr uptake measured by [^14^C]Cr uptake assay. Coincubation with 10 μg/ml CHX to inhibit protein synthesis did not affect Cr uptake in response to low level Cr, but increased Cr uptake by 33% in cells exposed to 5 mM Cr, with this effect plateauing at 3 h. Figure 1*A* shows the mean of four independent experiments. These findings confirm that new protein is synthesized in response to high Cr concentration ([Cr]), which then acts to limit Cr uptake (*P* < 0.0001 by 2-way ANOVA). These findings are reflected in total intracellular creatine levels ([Cr]_i_), with 3 h exposure to 250 μM Cr resulting in a 31% increase in [Cr]_i_ over untreated controls that was not significantly altered in the presence of CHX. In contrast, exposure to 5 mM Cr increased [Cr]_i_ accumulation by 80% (from 24.69 ± 2.07 to 44.50 ± 2.4 nmol/well; *P* = 0.008; Fig. 1*B*), with a further increase of 37% (to 53.61 ± 0.88 nmol/well) of control values in the presence of CHX (*P* = 0.026 for 5 mM Cr with and without CHX). This suggests that exposure to saturating levels of Cr results in synthesis of an unknown protein that acts to put a brake on further Cr uptake. When cells were analyzed for total [Cr], Cr values were not normalized over protein, since protein concentration was homogeneous throughout all wells, at 3.5 mg/ml throughout all of our experiments. Therefore, Cr values are reported as “nanomole per well.”

#### Effects of saturating [Cr] on global gene expression in vitro.

To identify the inhibitor of Cr uptake, 3T3-CrT cells were exposed to 5 mM [Cr] for 3 h before RNA isolation and global gene expression analysis. The array results were obtained using Illumina Beadstudio and analyzed to reveal 1,015 genes that were altered because of saturating [Cr], out of the total 45,000 probes on the gene array Illumina Beadchip. Of these, 10 genes were significantly altered when the *P* value threshold was set at ≤0.05 (Table 2). Txnip was the only gene to be upregulated in the presence of 5 mM Cr, with mRNA expression increased to 146% (*P* = 0.013) compared with untreated controls set at 100%. Txnip was therefore taken forward as a candidate CrT inhibitor, to undergo further verification in Cr uptake, gene and protein expression studies.

**Table 2.** Significantly changed genes Illumina Gene Array experiment

Gene ID	Fold Change	Adj *P* Value	Gene Symbol	Definition
5860358	0.6351	0.0107	*Vegfa*	Vascular endothelial growth factor A transcript variant 1
*2230730*	0.6586	0.0107	*Gjb2*	Gap junction membrane channel protein β_2_
*780554*	0.6564	0.0136	*Sema7a*	Semaphorin 7A
*70341*	1.4599	0.0136	*Txnip*	Thioredoxin-interacting protein 2
*6520075*	0.7039	0.0234	*Ier3*	Immediate early response 3
*3130059*	0.6857	0.0234	*Lif*	Leukemia inhibitory factor transcript variant 2
*20612*	0.6842	0.0234	*Egr3*	Early growth response
*4050762*	0.6796	0.0243	*Serpinb2*	Serpin peptidase inhibitor clade B (ovalbumin) member 2
*3840040*	0.7195	0.0414	*Grasp*	General receptor for phosphoinositides 1 associated scaffold protein
*7050138*	0.7348	0.0414	*Gfod1*	Glucose fructose oxidoreductase domain containing 1

3T3-CrT cells were incubated in the presence of 5 mM creatine for 3 h before analysis of total RNA using a global gene array approach with Illumina Beadchips. Saturating Cr concentration led to upregulation of Txnip by 46% compared with untreated cells (*n* = 3 each treatment; *P* = 0.013). Adj P value, *P* value after multiple corrections test.

#### Verification of increased Txnip expression by high [Cr].

**Fig. 2.**
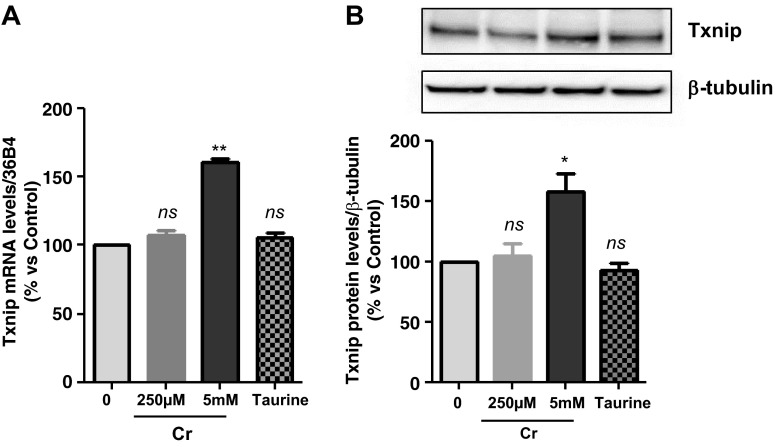
*A*: verification of gene array result by qRT-PCR. Cells were exposed to either 250 μM or 5 mM Cr. While 250 μM Cr did not change thioredoxin-interacting protein (Txnip) mRNA (107 ± 3.7; *P* = 0.449), saturating Cr caused a rise in Txnip mRNA levels in agreement to the gene array observation (*n* = 3 from 3 independent experiments; 161 ± 2; *P* = 0.0016). Moreover, the induction of Txnip expression was specific for Cr and did not occur at 5 mM taurine (*n* = 3; 106; *P* = 0.5481). *B*: Txnip protein levels were elevated by 5 mM Cr, at 3 h (*n* = 3/treatment; 145 ± 11; *P* = 0.0139), whereas neither 250 μM Cr nor taurine changed protein expression. ns, Nonsignificant *P* > 0.05, *P < 0.05, and **P < 0.005.

The gene array result was verified by a targeted gene expression approach using qRT-PCR in the same total RNA samples used for the gene arrays and in additional RNA samples from three independent experiments. This confirmed a rise in Txnip mRNA levels by 61% (*P* < 0.001 when exposed to 5 mM but not to 250 μM Cr). To test whether Txnip was elevated because of Cr and not because of a generalized response to osmotic stress caused by exposure to millimolar concentrations, experiments were repeated with 5 mM taurine added to growth media instead of Cr. Taurine did not raise Txnip expression levels, suggesting specificity of the Txnip response to high Cr levels (*P* = 0.55; 1-way ANOVA, Dunnett's posttest) (Fig. 2*A*). It was further demonstrated by immunoblotting that, although 250 μM Cr does not alter Txnip protein, 5 mM Cr increased Txnip protein by 45% after 3 h, as a direct reflection of the Txnip mRNA upregulation (*P* < 0.01; 1-way ANOVA and Dunnett's posttest; Fig. 2*B*). Taurine did not alter Txnip protein, in line with the lack of effects on mRNA levels (Fig. 2*A*).

#### Silencing Txnip expression alters CrT activity.

**Fig. 3.**
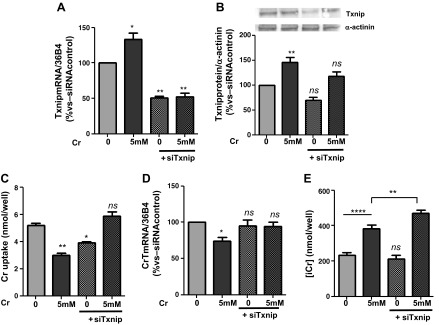
Effects of Txnip gene silencing in vitro. 3T3-CrT-HA cells were transfected with a small-interfering RNA (siRNA) oligonucleotide to inhibit mouse Txnip open-reading frame (siTxnip). *A*: basal Txnip mRNA levels were reduced to 50.74% (SE ±2.236) in the presence of siTxnip, and the Cr-mediated increase in Txnip transcript was abolished (52 ± 5; *P* = 0.018). *B*: there was a trend for reduced Txnip protein expression after siTxnip (70 ± 5), whereas the synthesis of new Txnip protein in response to 5 mM Cr was significantly attenuated (117 ± 9 vs. 145 ± 107 in the absence of siRNA; *P* = 0.018). *C*: Cr uptake is reduced following exposure to saturating levels of [Cr] for 3 h (3.007 ± 0.13), and this substrate inhibition is efficiently abolished by silencing Txnip (5.848 ± 0.34; *P* = 0.001). *D*: this effect was associated with preservation of CrT gene expression (1-way ANOVA, Dunnett's posttest vs. untreated; *P* = 0.0619). *C*: conversely, under basal conditions, siTxnip reduced Cr uptake via mechanisms unrelated to CrT gene expression (72 ± 1); *n* = 3/treatment was included in all experiments. *E*: intracellular Cr was raised by 65% (from 213 ± 15 to 383 ± 20 nmol/well) after exposure to 5 mM Cr for 3 h in vitro. When Txnip mRNA was reduced by siTxnip, the increasing effects of Cr were enhanced by a further 38% (to 470 ± 18 nmol/well; *P* = 0.0064). ns, Nonsignificant *P* > 0.05, *P < 0.05, **P < 0.01, and ****P < 0.001.

Cells were transfected with an siRNA oligonucleotide targeted against mouse Txnip open-reading frame. Forty-eight hours posttransfection, basal mRNA levels of Txnip were reduced to 50% of control samples without siRNA (“−siRNA control”; Fig. 3*A*). This was sufficient to abolish the increase in Txnip gene expression observed in response to 5 mM Cr (*P* < 0.0001). This pattern was also observed at the protein level. Following siTxnip transfection, there was a trend for reduced Txnip protein levels under baseline conditions (reduced by 30%), although this failed to reach statistical significance, which presumably reflects slow turnover of existing Txnip protein. However, the formation of new Txnip protein in response to Cr was significantly attenuated, increasing by only 17.3% (*P* = 0.011) compared with 45% (*P* = 0.014) in control cells, in good agreement with the extent of gene knockdown (Fig. 3*B*). The reduction in Txnip protein levels by 30% for zero Cr + siTxnip and then increase to 117% for 5 mM + siTxnip (Fig. 3*B*) are not significantly different from control, but they do occur in the opposite direction from one other, and the relative difference is 47 units, which is likely to be of some physiological significance reflected in the Cr uptake data. These results could also be taken to suggest that a certain level of Txnip is necessary to support baseline Cr uptake but that high Txnip expression results in CrT inhibition. As expected, prior exposure to high levels of Cr for 3 h impaired subsequent Cr uptake (i.e., substrate inhibition) by 39.8% (from 5 ± 0.15 to 3 ± 0.13 nmol/well) (Fig. 3*C*), and this was associated with reduced CrT gene expression (Fig. 3*D*). Under baseline conditions, gene silencing of Txnip reduced Cr uptake by 21% without altering CrT gene expression, suggesting a role for Txnip in maintaining CrT activity under normal physiological conditions. In contrast, with exposure to high [Cr], Txnip knockdown completely abolished substrate inhibition, preserving both Cr uptake and CrT gene expression at normal levels (Fig. 3, *C* and *D*). In 3T3-CrT cells that were exposed to 5 mM Cr for 3 h, intracellular Cr increased by 65% compared with untreated control (from 231 ± 15 to 383 ± 19 nmol/well), and by a further 38% (to 470 ± 18 nmol/well) when the cells were previously transfected with siTxnip (Fig. 3*E*). This rise in [Cr]_i_ when Txnip mRNA levels were blunted by 50% confirms the role of Txnip as a negative regulator of cellular Cr entry.

#### Myocardial intracellular Cr in vivo is linked to increased Txnip expression levels.

**Fig. 4.**
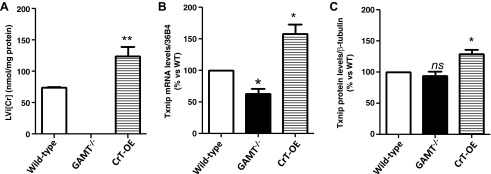
Relevance to myocardial tissue. *A*: LV Cr levels in mice overexpressing the Cr transporter (CrT-OE) were significantly higher than wild type (mean 124 nmol/mg protein vs. 74 nmol/mg, respectively; *P* = 0.0012), whereas Cr was confirmed to be undetectable in hearts from guanidinoacetate methyltransferase knockout (GAMT^−/−^) mice. *B*: GAMT^−/−^ had lower Txnip transcript levels than wild-type littermates (60 ± 7; *P* = 0.021). In contrast, CrT-OE showed higher levels of Txnip transcript compared with the wild type (158 ± 15; *P* = 0.0397). *C*: protein expression of Txnip was not statistically different in GAMT^−/−^ compared with the wild type, whereas CrT-OE had a 29% increase in protein (*P* = 0.012). Wild-type *n* = 10; GAMT^−/−^ *n* = 5; CrT-OE *n* = 8. Statistical symbols on graphs correspond to nonsignificant (ns) *P* > 0.05, *P < 0.05, and **P < 0.005.

To determine whether changes in Txnip expression levels are relevant to cardiac tissue, two mouse models of altered cardiac [Cr] were used. First, GAMT^−/−^ mice, which are Cr deficient, showed LV Txnip mRNA levels that were 35% lower than wild-type littermates (*P* = 0.021; Fig. 4*B*). However, this did not result in altered levels of Txnip protein (Fig. 4*B*). Transgenic mice with cardiac-specific CrT overexpression had elevated myocardial Cr levels (mean 124 nmol/mg protein compared with 74 nmol/mg protein in WT hearts), which was associated with markedly raised Txnip gene expression compared with wild types (by 58%; *P* = 0.0397) and GAMT^−/−^ (by 90%) (Fig. 4*B*). CrT-OE mice had 29% higher Txnip protein expression compared with the wild type (SE ±6.631; *P* = 0.012) (Fig. 4*C*).

## DISCUSSION

This study confirmed that exposure of cells to millimolar levels of Cr results in reduced Cr uptake (substrate inhibition) and that this is dependent on the synthesis of new protein (8, 19). However, the identities of these protein(s) were unknown. We therefore took a nonbiased gene array approach that identified *Txnip* as the only gene to be upregulated under these conditions, and this was confirmed at the protein level both in vitro and in vivo. In particular, in vitro knockdown of *Txnip* abolished the downregulation of Cr uptake and gene expression in response to high Cr levels, implicating Txnip as a mediator of substrate feedback inhibition.

However, our data also suggest that Txnip regulation of CrT is not straightforward. When Txnip was knocked down under basal conditions, we observed a reduction in cellular Cr uptake, suggesting that a certain level of Txnip expression is necessary to maintain normal CrT activity, in contrast to high levels of expression being associated with CrT inhibition. These effects appear to be driven by different mechanisms since inhibitory effects are associated with reduced CrT gene expression, whereas regulation under basal conditions was independent of CrT gene expression. It has previously been demonstrated that translocation of the CrT to/from the plasma membrane is another important factor in controlling Cr uptake (8).

Txnip is a redox-sensitive α-arrestin with pleiotropic cellular actions, making it an interesting candidate to mediate these effects. For example, Txnip is localized in the cytoplasm and has been shown to translocate to both the nucleus and plasma membrane (46, 47) and is therefore well placed to interact directly with either the CrT or with transcription. It was initially described for its ability to bind and inhibit the antioxidant properties of thioredoxin by forming a disulfide bond at cysteine-247 (13, 25). This interaction suggests an important role for Txnip in the regulation of redox signaling (32). It is increasingly recognized that the activity of many key regulatory proteins may be modified by *S*-nitrosylation (SNO), a nitric oxide-driven, posttranslational modification that converts a protein Cys thiol to an *S*-nitrosothiol. This process is dependent on the dynamic balance between SNO and denitrosylation, with the main physiological mediators of enzymatic denitrosylation being the *S*-nitrosoglutathione reductase and thioredoxin systems (3). A recent proteomic study of SNO in murine myocardium identified 951 unique protein targets, including 45 proteins involved in molecular transport (14). Txnip may exert its effects on CrT by regulating SNO, providing a promising line for future investigation. Previous studies have identified other posttranslational modifications resulting in altered CrT activity, for example, via phosphorylation in response to starvation and sepsis in vivo (44, 51), and via *N*- glycosylation sites, which influences both altered surface trafficking and transporter function (38). The ability of Txnip to modify these processes is also worthy of further study.

Txnip has reported roles in many critical cellular functions, including inhibition of cell cycle (6; reviewed in Ref. 45) and glucose metabolism (33). There are interesting parallels with our current study in that Txnip is upregulated by high glucose in diabetic tissues (28, 33) and has been shown to regulate both insulin-dependent and -independent pathways of glucose uptake (28). The mechanism for modulating glucose homeostasis remains to be fully elucidated, but studies in Txnip knockout mice suggest it occurs independently of thioredoxin activity or cellular antioxidant capacity (49). Furthermore, a large number of proteins are differentially expressed in the heart as a consequence of Txnip deletion, despite no measureable effect on thioredoxin activity under baseline conditions. The overall effect is to reduce mitochondrial function while enhancing anaerobic metabolism, suggesting that Txnip is a key modulator of myocardial energy homeostasis (48).

One limitation of our study is that the mouse models used to demonstrate relevance to in vivo myocardium represent extremes of low and high Cr levels that are not observed under normal physiological (or even pathophysiological) conditions. Our findings on GAMT^−^/^−^, which lack myocardial Cr, indicate that Txnip is not a regulator of Cr uptake in cases of low, but only in response to raised, intracellular Cr levels, as shown in the CrT-OE mice. Therefore, whether the Txnip pathway is involved in day-to-day regulation in response to small fluctuations in myocardial Cr is a moot point and may be better addressed once the molecular basis of the Txnip-CrT interaction is understood. Nevertheless, this aspect of our study provides important insight by demonstrating that blocking the Txnip-CrT interaction is a plausible in vivo target where the goal is to force Cr levels higher than the physiological norm. This is an attractive strategy since we have recently demonstrated that elevation of myocardial Cr by up to 100% above wild-type values protects the heart against subsequent ischemia-reperfusion injury (21).

In conclusion, we have identified Txnip as a novel negative modulator of Cr uptake in vitro, which is also relevant to myocardial Cr regulation in vivo. Further investigations of the molecular mechanisms of this interaction are merited, e.g., via redox signaling, posttranslational modification, or intracellular translocation of the Cr transporter, with a view to identifying new approaches for pharmacological control of cellular Cr homeostasis.

## GRANTS

This work was funded by the British Heart Foundation (BHF) Programme Grant RG/10/002/28187 and by the BHF Centre of Research Excellence, Oxford. N. Sahgal is supported by a Wellcome Trust Grant [090532/Z/09/Z].

## DISCLOSURES

No conflicts of interest, financial or otherwise, are declared by the authors.

## AUTHOR CONTRIBUTIONS

Author contributions: S.Z., S.N., and C.A.L. conception and design of research; S.Z., T.R., L.S.-M., R.C., D.J.M., and P.J.O. performed experiments; S.Z., T.R., N.S., and R.C. analyzed data; S.Z. and C.A.L. interpreted results of experiments; S.Z. prepared figures; S.Z. drafted manuscript; S.Z., T.R., N.S., L.S.-M., S.N., and C.A.L. edited and revised manuscript; S.Z., T.R., N.S., L.S.-M., R.C., D.J.M., P.J.O., S.N., and C.A.L. approved final version of manuscript.
